# Some Familiar Practical Methods in Bacteriology

**Published:** 1887-07

**Authors:** E. O. Shakespeare

**Affiliations:** Ophthalmic Surgeon and Pathologist to the Philadelphia Hospital


### Art. XXIV.—SOME FAMILIAR PRACTICAL METHODS IN BACTERIOLOGY.

BY E. O. SHAKESPEARE, A. M., M. D.

*Ophthalmic Surgeon and Pathologist to the Philadelphia Hospital.*

The practical importance of bacteriological research, in its bearing upon the etiology and diagnosis of infectious diseases, is at length beginning to be properly appreciated in this country, where, perhaps more than in any other, scientific questions are viewed from the stand-point of utility.

As such researches necessarily consume much time, even under the most favorable circumstances, a few words concerning some of the practical methods which actual experience has proved to be satisfactory may be the means of saving some trouble and time to those whose occupations leave them little of the latter to lose.

1st. As to methods of staining. It is now unquestioned that the satisfactory staining of bacteria is a *sine qua non* in their preparation for study under the microscope. The few remarks which will be made under this head will be limited to a brief explanation of the mode of preparation and use of some of the most generally applicable coloring fluids.

*a. Method of staining tubercle bacilli.*—I have found that the Koch-Erlich-Rindfleisch rapid method has constantly given satisfactory results, both as to reliability and rapidity. With it, the examination of sputum or other fluid or semi-fluid material for the presence of the tubercle bacilli, requires no more time and trouble than the examination of urine for tube casts, indeed, not nearly so much of either, as the time required for the settlement of the solid particles suspended in the urine is often considerable.

As soon as the fresh material suspected of containing tubercle bacilli is obtained, it may be at once spread upon a cover-glass as the first step in the process of examination. A word just here as to the collection of this fresh material. Of course, the vessel in which it is collected and kept, should be chemically clean, that is, sterilized, preferably by means of high dry heat, say 150° C., for an hour. In the case of sputum, it is best that the expectoration be ejected first into a broad shallow vessel, such as a saucer. Then the more solid and consistent portion of the sputum, freed as much as possible from mucus and saliva, should be transferred to the vessel in which it is to be transported to the place of examination. The material should be prepared for examination as fresh as possible, for it should be well understood, that not only do the common bacteria grow and multiply very rapidly in ordinary sputum, which is kept, but even the usually slowly developing tubercle bacilli increase in numbers.

In the case of sputum, a thin film of the thick, opaque portion is spread upon a cover glass. In the case of a supposed tubercle, removed from the tissue, the lungs for instance, it is to be crushed, moistened with a drop of sterilized distilled water, and the grumous fluid spread on the cover glass in as thin a film as possible. Suspected pus, urine, or any other fluid or semi-fluid matter, is to be spread upon the cover glass in a similar manner. The film thus prepared, is now allowed to dry *spontaneously* in the air of the room, *without extra heat.* As soon as it is thoroughly dry, the cover glass, with the film uppermost, is quickly passed through the flame of a spirit lamp, three times, in order to fix the film, so that it may not be washed away during the staining process. It is preferable to hold the cover glass by the edges, between the thumb and index finger, in order that the sensations may guard against the over-heating, which is likely to occur if a pair of forceps be substituted for the fingers, inasmuch as over-heating seriously interferes with the staining of many forms of bacteria.

For the staining, have, in a row, 5 good size watch glasses: in no. 1, place strong nitric acid, 1 part, distilled water 4 parts; in no. 2, place alcohol, 75 per cent.; in no. 3, place alcohol, 95 per cent.; in no. 4, place aqueous solution of methyl blue, so dense as to be just transparent when in the watch glass; in no. 5, place distilled water. Have another watch glass, three-quarters filled with the following coloring fluid:

| | | | | | |
|---|---|---|---|---|---|
| Of a | saturated | aqueous solution of | anilin oil, | 100 | parts. |
| " | " | alcoholic " | fuchsin, | 13 | " |
| " | | strong alcohol, | | 10 | " |

This mixture is to be filtered, before using, and should be freshly made.

Now float the cover glass, *film down*, upon the surface of the fuchsin mixture. Heat the mixture over a spirit-lamp, until bubbles begin to appear. Remove the heat at once, and allow the cover glass to remain in the hot fluid from two to four minutes.

Then seize the cover glass with forceps, immerse it for a second or two in watch glass No. 1, still holding on with the forceps; immediately immerse it in watch glass No. 2, until the excess of the red color has been washed from the film; then transfer it to No. 3, moving it back and forwards in the alcohol until the film becomes gray and has lost all trace of the red color; now float it, *film down*, upon the blue fluid in No. 4, and allow it to remain one or two minutes. Wash off the excess of color in watch glass No. 5, and stand the cover glass on edge upon bibulous paper to dry, preparatory to permanent mounting in Canada balsam. The preparation may, however, be examined temporarily, before permanently mounting, simply by mounting it in water.

The whole time required for the process of staining, from the time the unstained film is prepared till the stained film is stood up to dry, is only six to seven minutes.

I prefer the red for the tubercle bacillus stain, with the methyl blue for the contrast color, to the usual combination of methyl-violet and Bismark brown, because the common bacteria usually do not stain intensely with the brown, whilst, on the contrary, they stain well with the blue.

In preparing sections of tissues for staining, they should be cut as thin as possible, and stained as soon as cut. Leaving them in alcohol over night makes it very difficult, and oftentimes quite impossible to stain the bacteria. This is a remark which applies to sections of any tissue in which bacteria of any kind are to be sought for.

The staining of sections for demonstration of tubercle bacilli cannot be satisfactorily done by the short method above described. The sections should remain, at least over night, better still, twenty-four hours, in the mixture of fuchsin, and *without heating.* The sections are then subjected to the same treatment as above described for the cover glass film.

Either of the two above named coloring fluids can be used for staining ordinary bacteria in cover glass films. And to prepare bacteria for staining, if not in sections, it is a general rule to spread out the fluid containing them in as thin a film as possible, on a thin cover glass, and then fix the film by heat in the manner above described.

In most cases, except in staining for the tubercle bacillus, bacillus leprae and the bacillus of Lustgarten, the following treatment of the cover glass film is applicable:

Place a drop or two of the selected color fluid upon the film, and invert it upon a glass slide. Allow the fluid to remain thus in contact with the film one to five minutes, according to circumstances. Then with a pipette put a drop of distilled water on one side, at the edge of the cover glass, and on the opposite side place a piece of bibulous paper in contact with the edge. The color fluid is thus drawn out and is replaced by the distilled water. A little further irrigation completes the washing out of the superfluous coloring matter, and the preparation is now ready for immediate temporary examination, or for drying and permanent mounting in balsam.

There are a few species of bacteria besides those of tubercle, lepræ and syphilis, which are somewhat difficult to stain intensely. Among them are those of glanders and typhoid fever. I have found the method of Loeffler of Berlin quite satisfactory for demonstration of the glanders bacillus. It is as follows: The staining fluid consists of:

| | |
|---|---|
| Methyl blue (saturated alcoholic solution), . . | 34 parts |
| Slightly alkaline water, (1 part soda to 10,000 parts aq. dist.) . . . . . . . . | 66 parts |

Stain the film with this in the usual manner. Then wash out the superfluous color in the manner of irrigation, as above described, but, instead of a drop of pure distilled water, at first use a drop from a distilled water which has been acidulated with a drop or two of acetic acid. Finally, wash this acidulated water out well with pure distilled water.

Sections of tissues should be allowed to remain in the above staining fluid from twenty minutes to an hour, or two. They should be washed in slightly acidulated water in order to discharge the superfluous color, and to fix the color in the nuclear elements.

For the demonstration of the typhoid bacillus, especially in sections of tissues, I have found the method of Gaffkey, of Berlin, work very well. It consists chiefly in using, as a coloring fluid, a saturated watery solution of blue or violet, and avoiding the use of *acidulated* water in washing out the excess of color.

(*To be continued.*)

